# A randomized controlled trial to evaluate utilization of physical activity recommendations among patients of cardiovascular healthcare centres in Eastern Slovakia: study design and rationale of the AWATAR study

**DOI:** 10.1186/s12889-018-5349-1

**Published:** 2018-04-04

**Authors:** Aurel Zelko, Alena Bukova, Peter Kolarcik, Peter Bakalar, Ivan Majercak, Jana Potocnikova, Sijmen A. Reijneveld, Jitse P. van Dijk

**Affiliations:** 1 Institute of Physical Education and Sport, P. J. Safarik University, Ondavska 21, 040 11 Kosice, Slovakia; 2 Graduate School Kosice Institute for Society and Health, Faculty of Medicine, P. J. Safarik University, Trieda SNP 1, 040 11 Kosice, Slovakia; 3 Department of Health Psychology, Faculty of Medicine, P. J. Safarik University, Trieda SNP 1, 040 11 Kosice, Slovakia; 4 Olomouc University Society and Health Institute, Palacky University Olomouc, Krizkovskeho 8, 771 47 Olomouc, Czech Republic; 5 First Department of Internal Medicine, Faculty of Medicine, P. J. Safarik University, Trieda SNP 1, 040 11 Kosice, Slovakia; 6 Department of Community and Occupational Medicine, University Medical Center Groningen, University of Groningen, Hanzeplein 1, 9700 Groningen, RB Netherlands

**Keywords:** Exercise prescription, Digital exercise prescription tool, Cardiovascular disease

## Abstract

**Background:**

Guidelines on modifiable risk factors regarding cardiological patients are poorly implemented in clinical practice perhaps due to low health literacy. Several digital tools for improving lifestyle and behavioural intervention were developed. Our primary aim is to evaluate the effectiveness of a digital exercise prescription tool on the adherence to physical activity recommendations among patients with cardiovascular diseases.

**Methods:**

A randomized controlled trial will be realized in cooperation with Cardiovascular Health Centres in Eastern Slovakia. Patients recruited through their cardiologists, will be randomised at 1:1 ratio to the three-months’ experimental condition or control condition. The experimental group will receive standard lifestyle consultation leading to individually optimized prescription of physical activity. The control group will receive standard, usual-cardio-care lifestyle counselling, also in the domain of physical activity. The digital system will be used for optimized exercise prescription. The primary outcome is a change in the patient’s adherence to exercise recommendations. Data will be collected in both groups prior to consultation and after 3 months.

**Discussion:**

This study protocol presents background and design of a randomized control trial to investigate the effectiveness of a digital system-provide exercise prescription tool on the adherence to physical activity recommendations. An optimized exercise prescription that better reflects patient’s diagnosis, comorbidities and medication can have a significant impact on secondary prevention of cardiovascular disease. This trial can provide important evidence about the effectiveness of digital exercise guidance in everyday practice of cardiovascular healthcare.

**Trial registration:**

The study was registered on 1st November, 2017 and is available online at ClinicalTrials.gov (ID:NCT03329053).

## Background

The age-standardised prevalence rate of cardiovascular disease (CVD) among the Slovakian male population (9,403 per 100,000) is the highest within the EU countries, while among females the comparable prevalence rate, approaching 7000 per 100,000, is the second highest across the EU member states [1]. In response to these statistics, a number of action calls are nowadays proclaimed for better and more effective prevention of CVD [2–6]. Exercise prescription is an essential part of primary and secondary CVD prevention programs.

Increasing exercise is clinically efficient in blood pressure management. A systematic review and meta-analysis of randomized controlled trials focused on the effects of exercise intervention in normotensive and hypertensive patients confirmed that resistance [7] and endurance [8] training lowers the systolic and diastolic blood pressure in patients with CVD. Exercise-induced hypertension reducing effects were confirmed in young [9], middle-aged [10] and elderly [11–13] population and also in patients with low responsiveness to hypertension medical treatment [14, 15]. Physical activity (PA) is seen as an effective modulator of the endothelial function [16], which was recognized as a main trigger of the cardioprotective effects of exercise [17–20]. Regular exercise performed by middle-aged and older healthy men should prevent age-associated loss in endothelium-dependent vasodilation and restore endothelial activity to levels similar to those of younger men [21]. Moreover, PA was able to attenuate the deleterious effect of nitric oxide synthase gene polymorphism on the systolic blood pressure [22] and has shown to have positive impacts on glycaemic control [23], cholesterol profile [24] and lipoprotein subclass profile [25].

To ensure maximal effectiveness and safety of exercise interventions [26, 27], experts in the field of cardiovascular health are regularly updating the guidelines containing recommendations for PA of patients with CVD. Between 2004 and 2006, the European Society of Cardiology (ESC) published miniseries of position papers that summarized specific recommendations for PA of patients by pathology [28–33]. A three-part miniseries published by the ESC in 2012 was describing the importance of PA and exercise modalities in the management of cardiovascular health. This time, exercise recommendations were dedicated for the general population [34], individuals with cardiovascular risk factors [35], and individuals with CVD [36]. The latest upgrade of ESC’s guidelines for PA was done in the year 2016 [37].

Despite the strong emphasis on regular exercise during lifestyle counselling [38], in the EUROASPIRE IV (European Society of Cardiology survey on the lifestyle, risk factor and therapeutic management of coronary patients), more than 59% of the interviewed patients reported little or no PA [39]. To clarify these conflicting observations, we need take to account the ecological factors [40], the quality of communication between care-providers and patients, [41, 42] and the patient’s skills to access, understand and use of disease-specific instructions and recommendations [43, 44]. The latter domain is also labelled as the patient’s health literacy (HL). The HL level influences the patient’s use of healthcare, quality of disease management and health risk behaviours [45] and reflects patient’s proactivity in attempting to utilize health literacy skills [43]. Sufficient HL is significantly associated with improved health outcomes with a lower cost of care [45], while HL inadequacies can compromise patient safety, worsen management of a disease and increase the incidence of health conditions requiring intense medical assistance or hospitalization [46]. In older adults, inadequate HL was significantly associated with poor implementation of PA recommendations [47]. For improvement of patient’s accessibility and applicability of health-related knowledge in real life, we need to emerge that patients and health professionals cannot address these needs independently [48].

For cardio-clinicians nowadays, the available time is very limited to implement all parts of the long versions of disease-, comorbidity-, and medication-specific guidelines for prevention of CVD [28–37]. Behavioural interventions mediated by a digital tool may be a feasible route to improve cardiovascular-related medication adherence and clinical outcomes [49–51]. For the improvement of exercise prescriptions accessibility and applicability in the secondary prevention of CVD, a digital support system for optimized exercise prescription seems to be appropriate. In response to this request, the European Association of Preventive Cardiology developed and introduced the Exercise Prescription in Everyday Practice and Rehabilitative Training (EXPERT) tool [52]. In this “utilization of physical activity recommendations among patients of cardiovascular healthcare centres” (AWATAR) trial, we will assess whether the EXPERT tool can effectively increase the utilization of exercise recommendations among patients with CVD. The effects of such a digital intervention may further be moderated by HL. Therefore, we will try to confirm that patients with higher HL might benefit more from the digital tool prescription compared with patients with lower HL.

### The aims of the AWATAR trial are therefore

1. to determine and compare the utilization of exercise recommendations after exercise prescription according to digital training and decision support system as compared to exercise prescription following the standard informative procedure among patients with CVD.
2. to determine whether HL moderates the effect of exercise prescription according to the digital training and decision support system on the indices of exercise recommendations adherence among patients with CVD.

## Methods/design

### Trial design

To ensure complete reporting and sufficient replicability of our trial, we describe its design following the SPIRIT 2013 Statement [53] and create a schedule of enrolment, interventions, and assessments planned on the trial based on the SPIRIT template (Table 1). The AWATAR study will be the first randomized controlled trial (RCT) in which the utilization of exercise recommendations between the standard lifestyle informative procedure and the digital training and decision system informative procedure will be compared. Based on the reviewed literature, we hypothesize that a higher rate of adherence between the patient’s 3 months exercise report and patient’s digital tool exercise prescription will be observed in the experimental group compared with a three-months waiting group that receives care-as-usual. The trial protocol was approved by Human Research Ethics Committee of the Pavol Jozef Safarik University (approval no. PJSU-V-0825/17–1).

**Table 1:** SPIRIT schedule of enrolment, interventions and assessments for experimental and control group in the AWATAR trial

	Study period		
Timepoint	Enrolment T_0_	Between T_0_ and T_1_	At three months T_1_
Enrolment:			
Eligibility screen	X		
Informed consent	X		
Allocation	X		
Intervention (either intervention under study or CAU)		#	
Assessments:			
Questionnaire on Adherence to recommendations	X		X
PA questionnaire	X		X
Anthropometry	X		X
Clinical data	X		X
Health literacy	X		X
Lifestyle variables	X		X
Socio-demographic data	X		X
Feasibility	X		X

(X - application of procedure in both experimental and control group; # - application of EXPERT tool in experimental group or application of standard informative procedure in control group)

### Participants

All potentially eligible participants from the cooperating Cardiovascular Health Centres (CHCs) will be screened and selected according the inclusion criteria exclusively through their cardiologists and receive written information about the trial. Within a week they will be contacted by phone to be provided by additional details about the trial and to confirm their availability to participate in the study. After telephonic agreement to participate in the study, they will be invited to an introductory session with the treating cardiologist. The cardiologist will review the inclusion criteria as indicated above and the exclusion criteria and discuss with the patient further study details. At a final formal step, a written informed consent form will be obtained from the participants. Following the introductory session, the baseline assessments of participants will be made.

### Sample size

The trial has been designed to detect an effect size of 0.33 for the intervention under study compared to care-as-casual, regarding the primary outcome of agreement between patient’s reported and prescribed exercise indices (type, duration, intensity and frequency - variables summarized in Table 4), with a power of 80% at an α level of 0.05 (two-tailed). An effect size of 0.60 has previously been shown to be realistic [50] and has sufficient clinical relevance. This requires 150 participants in each group, i.e. a total effective sample size of 2 * 144 = 288 participants. Based on the experience with similar projects completed at the CHCs, the expected attrition rate is 25%. This implies that almost 400 participants should be recruited.

### Inclusion criteria

The study aims at assessing the effects in routine care, i.e. for a wide age range and a number of cardiovascular diagnoses on inclusion criteria. To be eligible to participate in the AWATAR trial, patients have to meet following criteria:

- age between 40 and 75 years-old;
- ability to provide informed consent based on information about perceived health benefits and risks connected with participation in the study from the treating cardiologist;
- willingness to accept randomization and participation in the assessment procedures;
- presence of one or more below listed cardiovascular diagnosis (Table 2) at the recruitment moment of participants.

**Table 2:** Inclusion criteria: present cardiovascular diagnosis at the recruitment moment of participants

Cardiovascular diagnosis	
coronary artery disease (both, with or without percutaneous coronary intervention)	
coronary artery bypass graft surgery, or endoscopic traumatic coronary artery bypass graft surgery	
compensated heart failure (with preserved or lowered left ventricular ejection fraction)	
cardiomyopathy	
intermittent claudication	
implantable cardioverter defibrillator or pacemaker	
ventricular assist devices	
heart transplantation	
valve disease or surgery	
congenital heart disease	
non-severe pulmonary hypertension	

### Exclusion criteria

After the participants’ inclusion, specific exclusion criteria regard minimization the risk of (sudden) exercise-induced cardiac events. Exclusion criteria include the presence of specific CVD problems that significantly increasing risks of sudden cardiovascular events, also identified as potential contraindications of PA on cardiovascular patients. These are summarized in Table 3-List A regarding specific cardiovascular conditions and List B regarding non-CVD specific exclusion.

**Table 3:** Exclusion criteria: presence of specific cardiovascular conditions and non-specific exclusion criteria

List A	Presence of specific cardiovascular conditions
	uncontrollable hypertension
	decompensated heart failure
	severe and symptomatic aortic stenosis
	uncontrolled arrhythmia
	severe pulmonary hypertension
	acute coronary syndromes
	acute myocarditis, endocarditis, or pericarditis
	aortic dissection
	Marfan syndrome
List B	Presence of non-CVD specific exclusion criteria
	malignancy (active cancer) or life-threatening disease
	bed-bound patient status
	participation or plan to participate in other study during trial execution
	plan to move during the trial execution

### Randomization and allocation

The AWATAR trial will be realized as a two arm, parallel-group, randomized controlled trial with an allocation ratio of 1:1. Participants will be randomly assigned, with equal probability to fall into each of two trial arms into: the experimental group (EG) and the 3 months waiting list control group (CG). For the allocation process, a computer-generated allocation sequence will be prepared by an investigator with no involvement in the trial. Allocation sequence will be stratified by the cardiovascular diagnosis of participants and will not be disclosed to the cardiologists or persons from the investigation team participating in the trial. This important blinding step will prevent assignment of patients into EG or CG groups according to someone’s personal preferences.

For the assignment process, we will prepare identical, sealed, opaque, stapled envelopes, sequentially numbered by generated allocation sequence. Each envelope will contain the written code (EG or CG) indicating experimental or control group. This code containing envelope will be drawn by one member of investigatory team (AZ). The nature of the intervention makes it impossible to ensure blinding of the investigatory team or participants in presented trial settings.

### Intervention

#### Experimental condition

After baseline assessments, patients in experimental group (EG) will undergo behavioural counselling. During the 30-min long consultation EG patients will receive standard, usual-cardio-care lifestyle, hypertension and cardiovascular instructions, except recommendations for physical activities (PA). Instruction s on this PA domain will be provided exclusively through the digital training and decision support (EXPERT) tool. They will receive this information immediately after the baseline assessments by a trained member of the investigatory team (AZ).

The EXPERT tool is an interactive digital training and decision support system for optimized exercise prescription in cardiovascular disease patients. The tool was developed by a working group connecting 33 experts in the rehabilitation of chronic internal diseases and three computer science experts from 11 European countries. During the construction of the system, clinical and rehabilitation experts reviewed all relevant and accessible recommendations, current clinical guidelines, studies and expert opinions in exercise intervention of cardiovascular patients. Specific exercise intervention goals were formulated for each different CVD, CVD risk factor and other chronic non-cardiovascular comorbidity.

The EXPERT tool was designed to provide optimized exercise prescription based on detailed overview of patient’s diagnosis, prognostics, complications and medication. The digital system requires data input in four steps, the data about patient’s clinical and cardiological characteristics (parameters), patient’s medication data and data summarizing patient’s adverse events registered during exercise testing. The EXPERT tool is able to generate exercise prescriptions even some of the relevant parameters are missing. In this case, unavailable data are not reflected in the prescription process and should negatively affect individualization and optimization rate of recommendations for patient in final.

After execution of the required data input, the EXPERT tool is ready to generate an exercise prescription (type of exercise) defining five regulatory parameters: 1) intensity and 2) frequency of the exercise, 3) duration of each exercise session, 4) duration of the training program, 5) feasibility of the strength training during the training program. Additionally, the prescription contains exercise contraindications and safety precautions for a certain type of exercise. The generated exercise prescription will be explained and discussed in details by a member of the investigatory team and will be printed in two paper copies for the patient. We will forward the EXPERT tool’s output in electronic form to patient’s email address. All outputs will be safely stored for replication or exercise prescription history review.

#### Control condition

Patients randomized to the (3 months waiting) CG will after baseline assessment receive standard, usual-cardio-care lifestyle, hypertension and usual cardiovascular instructions, so also in the domain of recommendations for physical activities. The exercise prescription process during consultation in the CG will be constructed in accordance with published ECS guidelines and recommendations for CVD secondary prevention [54]. The timing and mediator of behavioural counselling of the CG will be same as on the EG. Participants in CG will be informed, that after expiration of 3 months control period, and they will be offered to receive an identical digital-based (EXPERT) exercise prescription as patients in EG. Through the intervention all participants are instructed to maintain the standard treatment. Patients who declined to participate in the trial and those who will cancel participation during implementation of the trial will be asked to complete a short questionnaire to collect basic socio-demographic and behavioural data and explore the reasons for non-participation or non-continuation in the trial.

### Procedure

Assessments will be conducted at two time points – (prior to patient’s behavioural consultation and 3 months follow-up the consultation). Completion of measurements should take maximally 30 min. One member of the investigatory team (AZ) will perform all measures in the trial. A dedicated member will undergo a training session to ensure consistency of assessment protocol and validity and reliability of applied diagnostics instruments.

### Primary outcome measures

The *primary outcome* is exercise recommendations adherence as assessed by agreement between the patients reported and the prescribed indices of exercise. The indices are type, duration, intensity and frequency of exercise. Patient-reported type, duration, intensity and frequency of exercise will be assessed using a short self-report questionnaire (Table 4), reflecting the construction and definition of exercise prescriptions generated by the EXPERT tool. The questionnaire will be filled with assistance of an investigatory team member and he will ask participants to report regularity on exercise over the last year and describe in detail the type, duration, intensity and frequency of exercise realized over the last 3 months. Exercise recommendations adherence will be determined as agreement between indices of exercise prescription delivered by EXPERT tool and self-reported indices of exercise performed in monitoring period (3 months). In the follow up assessment, the last section of the questionnaire will be dedicated on the general evaluation of PA behavioural change (by the question: “Have you registered a change in your PA behaviour during the last three months?”; with options for answer: “Definitely/Partially/Only limited change/Not at all”).

**Table 4:** Measurement dimensions of exercise recommendations adherence and outcome variables

Measurement dimensions	Outcomes variables (independent data for last 3 months recall period)
Type of exercise	• Endurance training (walking, bicycling, swimming) • Strength training (free weight or resistance strength training, weightlifting) • Speed training (all short duration exercises with high intensity) • Anaerobic training (all exercises generating “lactate feelings”) • Sport games or mixed/other training
Duration of exercise (in one session)	• 0–10 min. • 10–20 min. • 20–30 min. • 30–45 min. • 45–60 min. • 60–90 min. • 90–120 min. • 121 and more min.
Intensity of exercise (typical intensity)	• Light Activity: an activity that does not cause you to ‘huff and puff’. • Moderate Activity: an activity that makes you breathe harder than normal, but only a little. • Vigorous Activity: an activity that makes you ‘huff and puff’.
Frequency of exercise (per week)	• Daily • 3–6 per week • 1–2 per week

### Secondary outcome measures

*Secondary outcomes* include anthropometry (body mass index, weight to height ratio), performance of leisure time physical activities, parameters of medical comorbidities (classification/severity) and clinical characteristics of the participants (systolic and diastolic pressure; blood total and low-density lipoprotein cholesterol concentration; fasting glycaemia; resting and peak heart rate during exercise testing; and peak oxygen uptake capacity).

*Anthropometry* Body mass index will be calculated (weight/(height)^2^) based on body height (m) and weight (kg) determined by a stadiometer and calibrated electronic scale, respectively. Waist-to-height ratio will be calculated by a standard formula (waist circumference/height), based on body height and waist circumference (cm) measured using a non-elastic tape measure at the narrowest point between the last rib and the iliac crest at the end of a normal expiration.

*Leisure time physical activities* To measure the prevalence of PA unqualified as sport-related or performance-oriented exercises (gardening, yard work, housework, carrying for another, home repairs) we will measure leisure time behaviours of participants via International Physical Activity Questionnaire – Short Form (IPAQ-SF). The IPAQ-SF is a validated and reliable questionnaire for assessment of frequency (days per week) and time (minutes per day) of PA realized during a past 7 days recall period in five domains: work, transportation, garden or yard, home, and leisure time [55]. The original English IPAQ-SF version was translated and cross-culturally adapted in several steps according to methodology established and described by the IPAQ developers group [http://www.ipaq.ki.se]. The Slovak IPAQ-SF version was subsequently reviewed by an expert and pre-tested [56, 57].

### Background measures

*Clinical data* included a list of patient’s CVD, CVD risk factors, other chronic non-cardiovascular co-morbidities, patient’s medication, systolic and diastolic blood pressure, blood total and low-density lipoprotein cholesterol concentration, fasting glycaemia, resting, and known patient’s adverse events registered during exercise testing. These data will be collected and reported by patient’s cardiologist and transferred from the patient’s medical record to the study file.

*The Health literacy* (HL) level will be assessed using The Health Literacy Questionnaire (HLQ) [58, 59]. This questionnaire comprises nine domains to provide a detailed profile of HL of populations, groups or patients of interest. The HLQ is divided into two parts which differ in response categories. Part 1 (domains 1–5) has 4 response categories rating the extent of agreement from 1 to 4. Part 2 (domains 6–9) has 5 response categories rating the level of difficulty from cannot do or always difficult (1), to always easy (5). Each domain was scored as the average of the item scores. The score ranges from 1 to 4 or 1 to 5 respectively, a higher score indicating higher level of health literacy. Cronbachs alpha in an earlier Slovak sample ranged from 0.73 to 0.84 among 9 HLQ domains [59].

Also, *lifestyle variables* and *socio-demographic data* will be collected via questionnaire developed for this study. The participants will provide standard information about age, education completed, marital status, number and age of descendants, living and work conditions and history, drinking and smoking habits.

#### Feasibility

Eligibility, compliance, attrition and safety rates will be registered during the recruitment and implementation of the study. The principal investigator, in cooperation with the cardiologists will register eligible patients out of all patients from the registries. Also, the number of those willing to participate out of all eligible patients will be recorded. During the intervention period, compliance and adherence data as well as the reasons for discontinuations will be collected. There are no contraindications, but specific measures incorporating partial body weight support may be necessary.

### Data analysis

All data analyses will be carried out using statistical software package IBM SPSS 22.0 or equivalent. Analyses will include standard descriptive statistics, Pearson chi-squared test for categorical variables, independent- and paired-sample tests of differences for continual variables (e.g. t-test/U-test, ANOVA) adjusted for baseline values. The agreement between indices of exercise prescription delivered by EXPERT tool and self-reported indices of exercise performed in monitoring periods will be tested using intra-class correlation test. Modelling of associations with possible adjustments will be conducted with regression analysis (linear and/or binary logistic). Analysis will be performed primarily based on intention-to-treat. In addition, a per-protocol analysis including only patients with no missing observations of the variable of interest will be performed. All tests will be two-tailed with statistical significance set at an α level of 0,05.

### Monitoring

The study will be supervised by a Data Safety and Monitoring Board consisting of one independent, internationally recognized clinical researcher (Assoc. Prof. Viola Vargova MD), one representative (cardiologist) from each cooperating Cardiovascular Healthcare Centre as an advisor and experts in health psychology (PK), exercise psychology (JP), trial management (PB), and exercise physiology (AZ). The Board will be the policy and decision-making authority of the trial. A principal investigator (AB) will be responsible for the organization of the research activities and communication with study patients, research co-operators and partners. Three investigator assistants will manage the central randomization (PB), project and ethics reporting (JP) and collection, protection, storing and processing of trial data (AZ). All assessments and exercise prescriptions in trial will be performed by one member of investigatory team (AZ).

## Discussion

In the present AWATAR trial the effectiveness of digital training and decision support system on patients with CVD will be investigated for the first time. Meeting PA recommendations is a challenging public health issue. Fulfilment of exercise recommendations is a key factor in CVD prevention [60, 61]. However, adherence rates among cardiovascular patients are deep below the expectation rates [39].

The strength of this trial is its study design, ideal for determining the effectiveness of an intervention in a real-world setting. In the Methods section, we specified a wide range of inclusion criteria to recruit the study population as similar as possible to the population on which the intervention tool is meant to be used. We tried to reflect the normal range of diversity in CVD severity, presence of comorbidities, and medication profiles seen in everyday clinical practice. The intervention (EXPERT) tool is an easy-accessible digital instrument and the outcomes are comprehensible for a range of users, including clinicians, patients, policy makers, and health commissioners. Also, the strong side of this survey is the use of the questionnaire items designed to specifically address the characteristics of exercise prescription for patients with CVD. The questionnaire items were developed by copping the exercise prescription variables defined by EXPERT tool.

The application of waiting control group design is weak side of the presented design. Based on similar studies, we expect that only one-third of patients randomized on control group will agree with control group settings and protocol. The high attrition rate in control group and the subsequently unbalanced drop-out rate between groups should affect internal validity of our study.

## Abbreviations

ANOVA: Analysis of variance
CG: Control group
CHCs: Cardiovascular Health Centres
CVD: Cardiovascular disease
EG: Experimental group
ESC: European society of cardiology
EU: European Union
EXPERT: Exercise prescription in everyday practice and rehabilitative training tool
HL: Health literacy
HLQ: Health literacy questionnaire
IPAQ-SF: International physical activity questionnaire – short form
PA: Physical activity
RTC: Randomized controlled trial
SPIRIT: Standard Protocol Items: Recommendations for Interventional Trials
